# Identification of quantitative trait loci for related traits of stalk lodging resistance using genome-wide association studies in maize (*Zea mays* L.)

**DOI:** 10.1186/s12863-022-01091-5

**Published:** 2022-11-01

**Authors:** Lifen Wu, Yunxiao Zheng, Fuchao Jiao, Ming Wang, Jing Zhang, Zhongqin Zhang, Yaqun Huang, Xiaoyan Jia, Liying Zhu, Yongfeng Zhao, Jinjie Guo, Jingtang Chen

**Affiliations:** 1 State Key Laboratory of North China Crop Improvement and Regulation, Hebei Sub-Center for National Maize Improvement Center, College of Agronomy, Hebei Agricultural University, Hebei, Baoding 071001 China; 2 College of Agronomy, Qingdao Agricultural University, Shandong, Qingdao 266109 China

**Keywords:** Maize, Stalk lodging resistance, Genome-wide association study, Quantitative trait nucleotides, Candidate gene

## Abstract

**Background:**

Stalk lodging is one of the main factors affecting maize (*Zea mays* L.) yield and limiting mechanized harvesting. Developing maize varieties with high stalk lodging resistance requires exploring the genetic basis of lodging resistance-associated agronomic traits. Stalk strength is an important indicator to evaluate maize lodging and can be evaluated by measuring stalk rind penetrometer resistance (RPR) and stalk buckling strength (SBS). Along with morphological traits of the stalk for the third internodes length (TIL), fourth internode length (FIL), third internode diameter (TID), and the fourth internode diameter (FID) traits are associated with stalk lodging resistance.

**Results:**

In this study, a natural population containing 248 diverse maize inbred lines genotyped with 83,057 single nucleotide polymorphism (SNP) markers was used for genome-wide association study (GWAS) for six stalk lodging resistance-related traits. The heritability of all traits ranged from 0.59 to 0.72 in the association mapping panel. A total of 85 significant SNPs were identified for the association mapping panel using best linear unbiased prediction (BLUP) values of all traits. Additionally, five candidate genes were associated with stalk strength traits, which were either directly or indirectly associated with cell wall components.

**Conclusions:**

These findings contribute to our understanding of the genetic basis of maize stalk lodging and provide valuable theoretical guidance for lodging resistance in maize breeding in the future.

**Supplementary Information:**

The online version contains supplementary material available at 10.1186/s12863-022-01091-5.

## Background

Maize (*Zea mays* L*.*) plays an important role in food security, feed provision, and fuel resources. Nevertheless, stalk lodging can lead to 5–20% maize yield loss annually worldwide [1]. Achieving high agricultural yields under different environmental conditions is a major goal of maize breeders. In low-density populations, the yield was improved by selecting taller plants to increase the biomass per plant. In high-density populations, the high yield was obtained by increasing the population density of selected medium height plants through the combination of reasonable panicle height coefficient and lodging resistance. Stable quantitative trait loci (QTLs) are particularly useful in marker-assisted selection [2]. Stalk lodging is a phenomenon whereby plants collapse from the upright state, a complicated and integrated quantitative trait caused by many factors, such as the quality of the stalk itself and the external environmental factors (e.g., climatic and soil conditions, planting density, fertilization and irrigation, pests and diseases) which cause irreversible damage to corn stalks and roots [1, 3]. Maize lodging can be divided into three types: root lodging, stem bending, and stem breaking [4]. Stalk lodging usually occurs at or below the ear node, which consequently influences the regular growth of the ear before harvest and the final yield of maize [5, 6]. Furthermore, grain yield per unit area is highly correlated to the plant’s adaptability to high crop density, but stalk lodging limits planting density and mechanized harvesting [7, 8]. Therefore, improving stalk lodging resistance in maize would benefit future breeding programs and agricultural production.

Stalk lodging resistance is correlated with stalk mechanical strength, hence this variable was used to evaluate lodging resistance in maize [9, 10]. Common methods to quantify the stalk mechanical strength include rind penetration, bending, breaking, and vertical crushing [4, 7, 11]. Most studies have found that the stalk rind penetrometer resistance (RPR) and stalk buckling strength (SBS) are important determinants of crop lodging resistance. Furthermore, RPR did not damage the stalk structure [12–14]. Compared with RPR, SBS is more closely correlated to stalk lodging under natural conditions, as stalk lodging happens in case of over-bending [15]. According to previous studies, we found that lodging occurs most frequently at flowering stage or a few weeks after flowering and the third or fourth internode of maize plants is extremely sensitive to stalk lodging in the field [6, 8, 13, 16]. Furthermore, Liu et al. [11] showed that the best period for evaluating stalk strength is the silking phase or stage after silking. The position of the stem lodging mainly occurs between the second and fifth internodes, especially in the third internodes and the fourth internodes above ground (FIAG) were significantly correlated with RPR and SBS [6, 8, 11, 17, 18]. In addition, with the increase of plant density, the length of the base nodes increased significantly, the diameter of the stems decreased significantly, and the content of cellulose, hemicellulose and lignin decreased, resulting in a decrease in the mechanical strength of the stems and an increased risk of lodging [19].

QTL mapping has been widely used in the study of various agronomic traits, including yield-related traits, which is a useful tool for analyzing the genetic structure of complex agronomic traits. In crop, QTL mapping on lodging have been gradually applied in sorghum, wheat, rice, especially in maize. For example, a linkage map with 129 SSRs markers was constructed by Hu et al. [6], and two, three, and two QTLs were detected for the maximum load exerted to breaking (F max), the breaking moment (M max) and the critical stress (σ max), respectively. Li et al. [12] identified seven QTLs associated with RPR in two maize recombinant inbred line (RIL) populations using 3072 single nucleotide polymorphisms (SNP) markers. Zhang et al. [17] identified 44 significant QTLs for SD, SBS, and RPR using the IBM Syn10 DH population in three environments.

The efficiency and accuracy of QTL mapping depend largely on the marker density, the variation range of phenotypes within the population, as well as the population size and type [20]. Genome-wide association study (GWAS) is a powerful tool for analyzing the genetic basis of complex traits. So far, GWAS has been used to analyze many agronomic traits such as plant height, leaf structure and yield-related traits [21–23], and other characteristics, i.e. In addition, some genetic studies on crop lodging have also been carried out using GWAS. On the contrary, although there are some GWAS reports on stalk lodging [13, 24], they are still relatively few, and the molecular mechanism of the variation of corn lodging-related traits is still poorly understood. High-throughput SNP markers have been widely used to identify genes controlling quantitative traits [25–28]. Genotyping by sequencing (GBS) is a relatively inexpensive method to obtain high-density markers for large populations taking the advantage of next-generation sequencing technologies [29–32].

In this study, an association mapping panel was genotyped by GBS. Based on this, association mapping was used to identify SNPs and excavate potential candidate genes on RPR, SBS, and morphological traits associated with stalk lodging resistance. The objectives of this study were to: (1) identify associated loci for RPR, SBS, and morphological traits of the stalk of maize; (2) ascertain stable SNPs and predict potential candidate genes in these regions; (3) dissect the genetic architecture of stalk lodging resistance-related traits.

## Results

### Phenotype analysis of the six lodging resistance-related traits

The phenotypes of all lodging resistance-related traits in the association mapping panel are shown in Table 1. The mean values of RPR, SBS, TID, and FID in the low plant density were higher than those in the high plant density. As for TIL and FIL, the mean values in the high plant density were higher than the mean values in the low plant density. For the six traits mentioned above, the skewness and kurtosis were less than 1, indicating that these traits followed a normal distribution. Furthermore, the coefficients of variation (CV) of these traits in the plant densities examined in this study ranged from 5.78–15.78% and 6.49–17.05%, respectively (Table 1).

**Table 1:** Phenotypic performance for related traits of stalk lodging resistance in the association mapping panel

Trait ^a^	Density ^b^	Mean ± SD	Range	Skewness	Kurtosis	CV (%)
RPR (N/mm^2^)	L	42.55 ± 5.70	29.61–60.78	0.43	0.24	13.39
	H	41.06 ± 4.68	29.74–54.51	0.15	-0.22	11.40
SBS (N/cm^2^)	L	429.08 ± 67.72	199,98–634.29	0.17	0.90	15.78
	H	354.04 ± 60.36	171.08–547.67	0.16	0.33	17.05
TIL(mm)	L	87.40 ± 9.10	65.60–110.39	0.04	-0.36	10.41
	H	90.50 ± 9.62	66.01–115.74	-0.03	-0.13	10.63
TID (mm)	L	17.55 ± 1.01	15.53–21.47	0.49	1.01	5.78
	H	16.73 ± 1.09	14.31–19.75	0.27	-0.07	6.49
FIL (mm)	L	103.90 ± 11.49	77.23–133.33	0.08	-0.47	11.06
	H	106.99 ± 11.04	79.92–135.88	-0.10	-0.47	10.32
FID (mm)	L	17.10 ± 1.00	14.96–20.09	0.39	0.58	5.85
	H	16.32 ± 1.08	13.95–19.29	0.22	0.10	6.60

^a^RPR, SBS, TIL, TID, FIL, and FID stand for rind penetrometer strength, stalk bending strength, third internode length, third internode diameter, fourth internode length, and fourth internode diameter, respectively

^b^L stands for low plant density, H stands for high plant density

ANOVA showed that the environment effects, density effects, genotype effects and interactive effects between the genotype and environment were both significant for six traits in the association mapping panel (Table 2). For the association mapping panel, the broad-sense heritability (*h*^*2*^_*B*_) of all traits in low and high plant densities ranged from 0.59 to 0.72 and 0.61 to 0.71, respectively (Table 2), suggesting that variations of stalk strength traits were mainly controlled by genetic factors.

**Table 2:** Analysis of variance (ANOVA) for related traits of stalk lodging resistance under two plant densities in the association mapping panel

Trait ^a^	*F*-value					*h*^*2*^_*B*_	
	Environment	Density	Genotype	Environment × Genotype	Density × Genotype	Low plant density	High plant density
RPR	477.91^**^	22.52^**^	11.36^**^	2.90^**^	1.73^**^	0.62	0.61
SBS	204.10^**^	432.13^**^	11.56^**^	2.01^**^	2.21^**^	0.67	0.65
TIL	47.41^**^	79.48^**^	10.76^**^	1.76^**^	1.12	0.66	0.70
TID	443.44^**^	87.55^**^	10.45^**^	1.78^**^	1.21^*^	0.59	0.67
FIL	310.40^**^	121.74^**^	11.21^**^	1.67^**^	0.79	0.72	0.71
FID	322.96^**^	86.36^**^	11.21^**^	1.84^**^	1.28^*^	0.61	0.68

^a^RPR, SBS, TIL, TID, FIL, and FID stand for rind penetrometer strength, stalk bending strength, third internode length, third internode diameter, fourth internode length, and fourth internode diameter, respectively

^*^Significant at *P* < 0.05

^**^Significant at *P* < 0.01

**Fig. 1:**
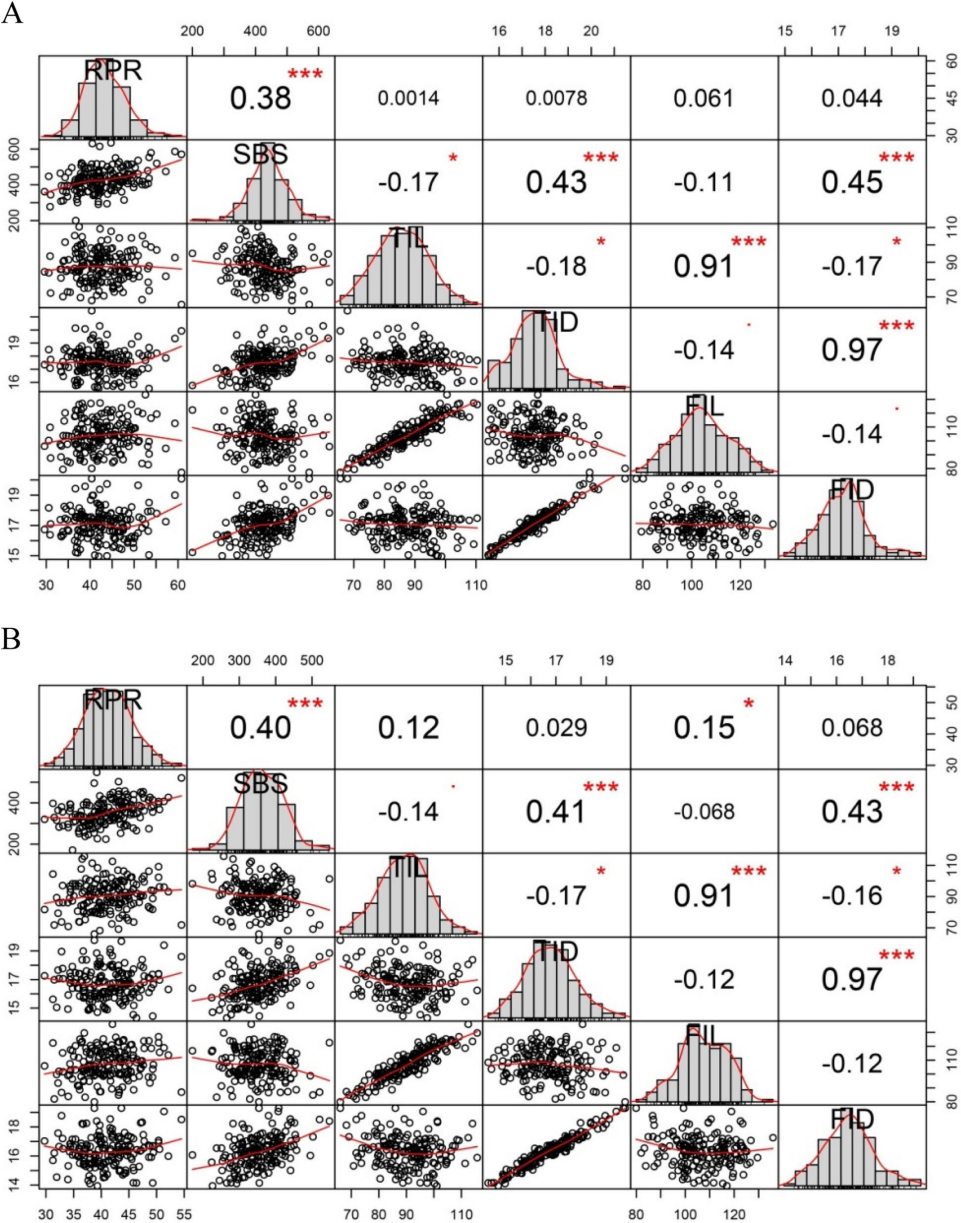
Correlation analysis of lodging resistance-related traits under two plant densities in the association mapping panel. A and B stand for low plant density and high plant density, respectively. * Significant at *P* < 0.05. ** Significant at *P* < 0.01

The results of the correlation analysis between the six traits of stalk strength at two densities for the maize inbred lines are shown in Fig. 1. In the correlation analysis, the consistency of all trait correlations between the two densities highly coincided with the results of GWAS. In addition, there was a strongly significant positive correlation between traits between SBS and RPR, SBS and TID as well as SBS and FID.

### GWAS for stalk lodging resistance related-traits

For RPR, a total of 29 significant SNPs were detected and located on chromosomes 1, 2, 3, 4, 5, 6, 7, 8, 9, 10 at all environments, which explained 11.10-16.07% of the phenotypic variation. For SBS, a total of 32 SNPs were detected across all environments, which explained phenotypic variation ranging from 9.29-17.69%. For other lodging resistance traits, the number of SNPs detected for TIL, TID, FIL and FID was 36, 53, 31 and 47, respectively, and accounted for phenotypic variation ranging from 12.31-20.72%, 11.23-18.50%, 13.96-23.59%, and 10.92%-17.44%, respectively (Table S1).

In total, 33 SNPs detected of different traits under same environment and density and explained phenotypic variation ranging from 11.23% to 20.70% (Table 3). Moreover, 2 significant SNPs for TIL were commonly detected across different environments, among which, Chr1_289271328 were identified in 2015BD, 2016BD and 2016SJZ at under high density and Chr2_54407952 were identified in 2016SJZ under low density and high density, with explanation of phenotypic variation range from is 14.97% to 18.14%. Moreover, one SNP, Chr2_233691764, was collocated for SBS, TID and FID on chromosomes 2 (Table 3).

**Table 3:** Important SNPs detected of different traits under same environment and density

Environment	Density^a^	Traits	SNP	Chr	Position (bp) ^b^	P-value	Allele	bin	PVE (%)
2015BD	L	SBS	Chr2_233691764	2	233,691,764	1.23E-05	C/G	2.09	13.55
		TID	Chr2_233691764	2	233,691,764	2.10E-05	C/G	2.09	16.43
		FID	Chr2_233691764	2	233,691,764	5.34E-05	C/G	2.09	14.90
		TID	Chr2_101115591	2	101,115,591	5.37E-05	A/G	2.05	15.25
		RPR	Chr6_113876033	6	113,876,033	4.13E-05	G/T	6.04	11.98
		TID	Chr6_129298262	6	129,298,262	4.52E-05	C/T	6.05	15.80
		TID	Chr6_129298294	6	129,298,294	4.67E-05	A/C	6.05	15.86
		FID	Chr6_129298262	6	129,298,262	2.86E-05	C/T	6.05	15.55
		FID	Chr6_129298294	6	129,298,294	3.58E-05	A/C	6.05	15.57
	H	TIL	Chr1_289271328	1	289,271,328	1.58E-05	C/T	1.11	18.14
		TID	Chr2_101115591	2	101,115,591	3.06E-05	A/G	2.05	16.94
		TIL	Chr2_157483756	2	157,483,756	5.14E-05	C/T	2.06	17.00
		FIL	Chr2_157483756	2	157,483,756	7.13E-06	C/T	2.06	20.70
		TID	Chr2_11053123	2	11,053,123	9.32E-05	A/G	2.02	15.54
		FID	Chr2_11053123	2	11,053,123	9.69E-05	A/G	2.02	14.53
		RPR	Chr6_113876033	6	113,876,033	4.24E-05	G/T	6.04	11.84
		TIL	Chr9_26826507	9	26,826,507	5.79E-06	C/T	9.03	19.48
		FIL	Chr9_26826507	9	26,826,507	4.94E-05	C/T	9.03	18.65
2015SJZ	L	TID	Chr1_159420156	1	159,420,156	3.03E-05	C/T	1.05	15.68
		FID	Chr1_159420166	1	159,420,166	4.18E-05	C/T	1.05	16.59
	H	TID	Chr1_251713297	1	251,713,297	3.36E-05	G/T	1.09	17.43
		FID	Chr1_251713297	1	251,713,297	9.44E-05	G/T	1.09	15.62
		TID	Chr2_209021682	2	209,021,682	4.15E-05	C/T	2.08	17.47
		FID	Chr2_209021682	2	209,021,682	9.88E-05	C/T	2.08	15.83
		TID	Chr2_4671519	2	4,671,519	9.11E-05	C/T	2.02	16.75
		FID	Chr2_4671519	2	4,671,519	3.25E-05	C/T	2.02	17.32
		TID	Chr4_79001631	4	79,001,631	5.25E-05	G/T	4.05	17.53
		FID	Chr4_79001631	4	79,001,631	7.59E-05	G/T	4.05	16.45
2016BD	L	TID	Chr1_256791485	1	256,791,485	4.71E-05	A/G	1.09	14.17
		FID	Chr1_256791485	1	256,791,485	1.82E-05	A/G	1.09	13.16
		TID	Chr4_175218919	4	175,218,919	7.09E-05	A/G	4.07	14.51
		FID	Chr4_175218919	4	175,218,919	7.45E-05	A/G	4.07	12.32
		FIL	Chr6_98760375	6	98,760,375	3.80E-05	C/T	6.03	15.70
	H	TIL	Chr1_289271328	1	289,271,328	1.58E-05	C/T	1.11	18.14
		FIL	Chr6_98760375	6	98,760,375	4.00E-05	C/T	6.03	16.05
		TIL	Chr6_147922112	6	147,922,112	1.22E-05	C/T	6.05	17.33
		FIL	Chr6_147922112	6	147,922,112	6.47E-05	C/T	6.05	15.17
2016SJZ	L	TID	Chr1_148452951	1	148,452,951	2.91E-05	G/T	1.05	15.33
		FID	Chr1_148452951	1	148,452,951	7.54E-06	G/T	1.05	16.33
		TID	Chr1_148452943	1	148,452,943	5.60E-05	C/G	1.05	15.29
		FID	Chr1_148452943	1	148,452,943	4.91E-05	C/G	1.05	14.89
		TID	Chr2_54407952	2	54,407,952	3.00E-05	C/T	2.05	15.50
		TIL	Chr2_216932638	2	216,932,638	3.76E-05	A/G	2.08	16.81
		FIL	Chr2_216932638	2	216,932,638	3.15E-05	A/G	2.08	16.12
		TIL	Chr2_216932653	2	216,932,653	6.35E-05	A/C	2.08	15.93
		FIL	Chr2_216932653	2	216,932,653	2.51E-05	A/C	2.08	16.09
		TID	Chr2_45966977	2	45,966,977	4.37E-05	C/G	2.04	15.39
		FID	Chr2_45966977	2	45,966,977	4.88E-05	C/G	2.04	14.68
		TID	Chr3_191764915	3	191,764,915	8.68E-06	A/C	3.07	16.38
		FID	Chr3_191764915	3	191,764,915	1.06E-05	A/C	3.07	15.49
		TID	Chr4_235448449	4	235,448,449	7.48E-05	A/G	4.09	14.25
		FID	Chr4_235448449	4	235,448,449	4.44E-05	A/G	4.09	14.24
	H	TIL	Chr1_289271328	1	289,271,328	7.74E-05	C/T	1.11	16.66
		TID	Chr2_54407952	2	54,407,952	2.19E-06	C/T	2.04	16.10
		FID	Chr2_54407952	2	54,407,952	1.57E-06	C/T	2.04	14.97
		TID	Chr2_54407976	2	54,407,976	4.52E-06	C/T	2.04	15.48
		FID	Chr2_54407976	2	54,407,976	5.07E-06	C/T	2.04	14.76
		TID	Chr2_12921336	2	12,921,336	5.30E-05	A/C	2.02	11.99
		FID	Chr2_12921336	2	12,921,336	3.41E-05	A/C	2.02	12.37
		TID	Chr2_12921363	2	12,921,363	9.33E-05	C/T	2.02	11.23
		FID	Chr2_12921363	2	12,921,363	4.23E-05	C/T	2.02	12.00
		TID	Chr3_8597909	3	8,597,909	5.23E-05	A/G	3.02	11.91
		FID	Chr3_8597909	3	8,597,909	4.64E-05	A/G	3.02	11.93
		TIL	Chr5_10438064	5	10,438,064	8.53E-05	C/T	5.02	16.56
		FIL	Chr5_10438064	5	10,438,064	7.21E-05	C/T	5.02	14.30
		TID	Chr5_125087688	5	125,087,688	4.98E-05	A/G	5.04	12.19
		FID	Chr5_125087688	5	125,087,688	4.32E-05	A/G	5.04	11.67

**Fig. 2:**
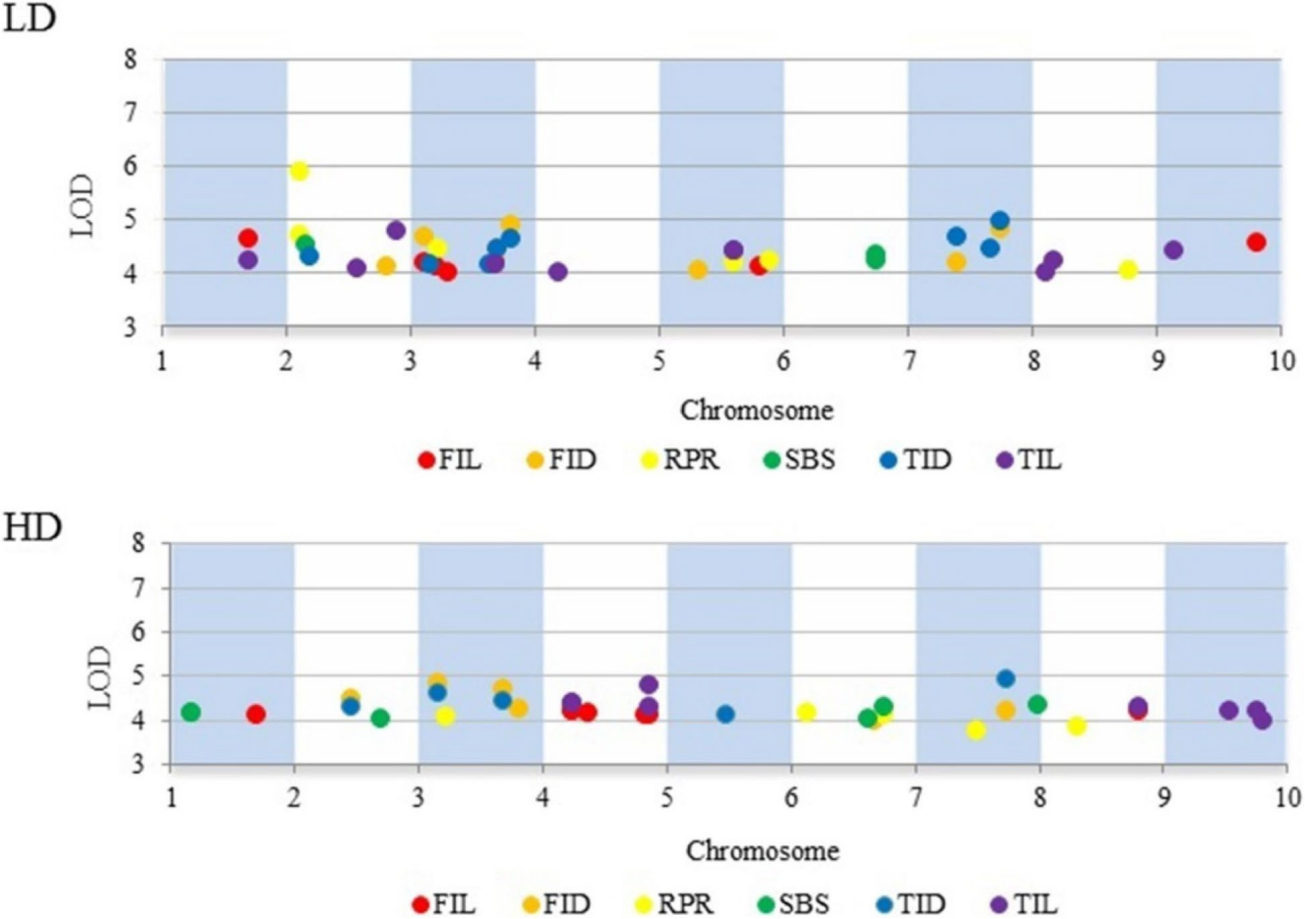
Stable SNPs were repeatedly detected in the two planting densities and the BLUP model, which were associated with six stalk lodging resistance-related traits. The significance threshold is –log10 (*P*-value) = 4.0. LD represent low plant density, HD represent high plant density, respectively. Purple represents third internodes length, Red represents fourth internode length, Blue represents third internode diameter, Orange represents fourth internode diameter, Yellow represents rind penetrometer resistance and Green represents stalk buckling strength, respectively

**Fig. 3:**
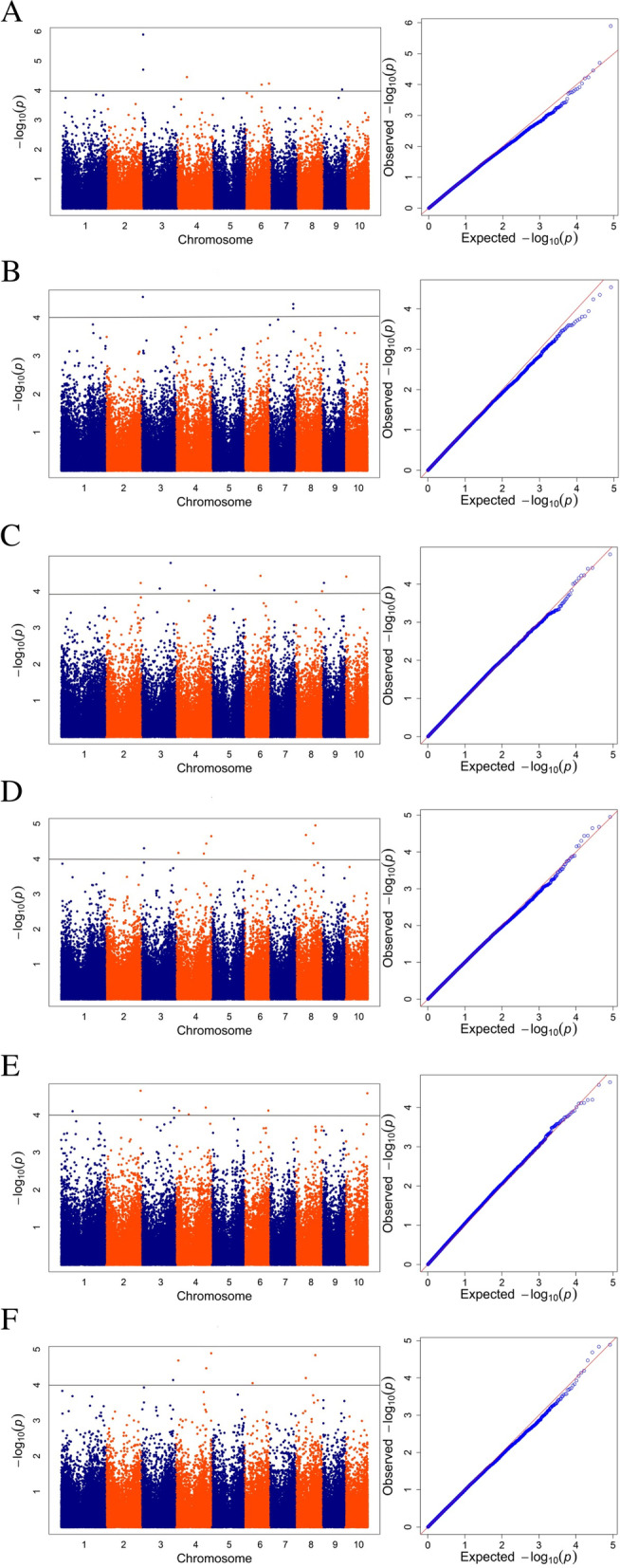
Manhattan plots and QQ plots for the six traits at the low plant density. **A** Rind penetrometer strength. **B** Stalk bending strength. **C** Third internode length. **D** Third internode diameter. **E** Fourth internode length. **F** Fourth internode diameter

**Fig. 4:**
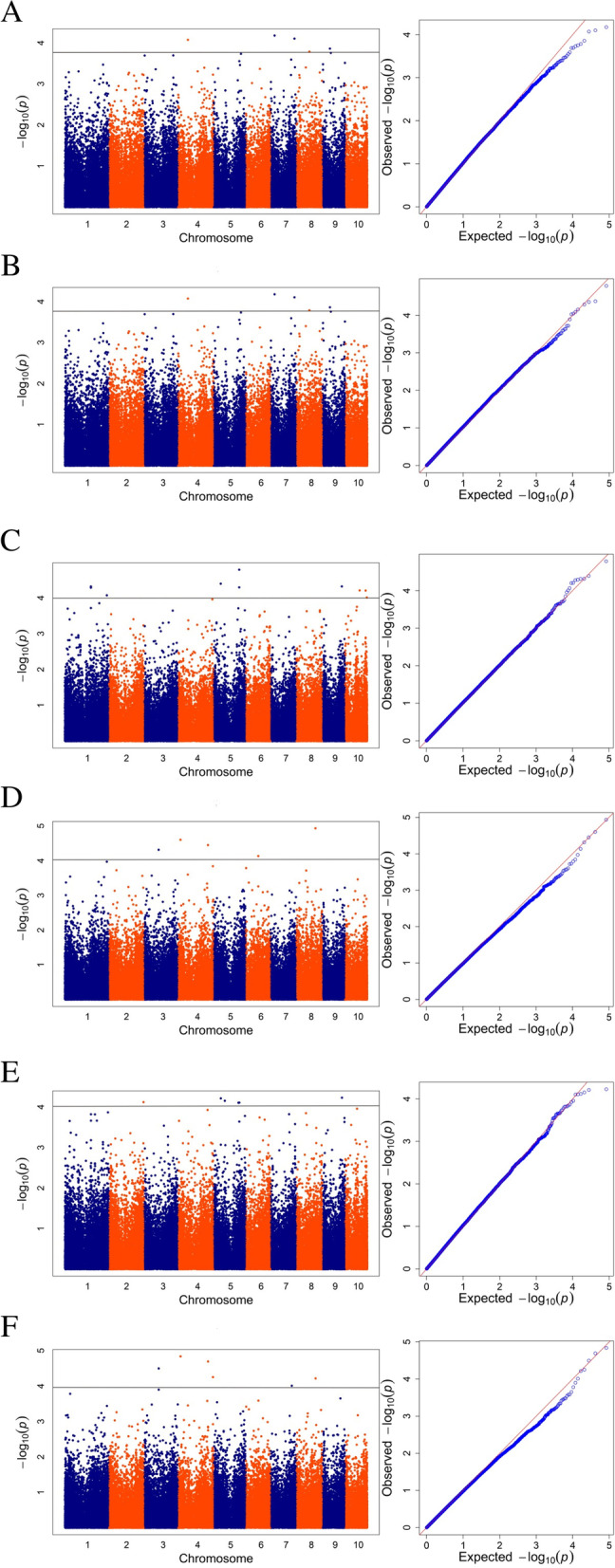
Manhattan plots and QQ plots for the six traits at the high plant density. **A** Rind penetrometer strength. **B** Stalk bending strength. **C** Third internode length. **D** Third internode diameter. **E** Fourth internode length. **F** Fourth internode diameter

To minimize the effect of environmental variation, the BLUP values were used to examine associations. In total, we identified the number of SNP for each trait by BLUP data, 6 for RPR, 3 for SBS, 10 for TIL, 8 for TID, 8 for FIL, 7 for FID at low plant density and 5 for RPR, 9 for SBS, 7 for TIL, 5 for TID, 7 for FIL, 6 for FID at high plant density (Fig. 2 and Table S2). The percentage of phenotypic variation explained by the identified SNPs (R^2^) for six traits ranged from 13.30 to 21.13% and from 10.10 to 21.01% at low and high plant densities, respectively (Table S2). The Manhattan plots and Quantile–quantile (Q-Q) plots between the six related traits of stalk strength at two densities are shown in Figs. 3 and 4. In addition, 14 important SNPs was detected of different traits at same density by BLUP value, which were located on chromosomes 2, 3, 4, 5, 8, 9 and 10 (Table 4).

**Table 4:** Important SNPs detected of different traits by BLUP value

Number	SNP	Traits	Density ^a^	Chr	Position(bp)^b^	Allele	bin	P-value	PVE (%)
1	Chr4_66017316	RPR	L	4	66,017,316	C/T	4.05	3.52E-05	16.10%
		RPR	H	4	66,017,316	C/T	4.05	8.47E-05	16.90%
2	Chr2_231360274	TIL	L	2	231,360,274	C/G	2.09	5.95E-05	19.56%
		FIL	L	2	231,360,274	C/G	2.09	2.27E-05	20.46%
3	Chr3_99647159	TID	H	3	99,647,159	A/G	3.04	4.86E-05	16.51%
		FID	H	3	99,647,159	A/G	3.04	3.19E-05	15.67%
4	Chr4_16211307	TID	L	4	16,211,307	A/G	4.03	6.74E-05	18.06%
		FID	L	4	16,211,307	A/G	4.03	2.05E-05	18.00%
		TID	H	4	16,211,307	A/G	4.03	2.51E-05	17.60%
		FID	H	4	16,211,307	A/G	4.03	1.45E-05	16.94%
5	Chr4_199957809	TIL	L	4	199,957,809	A/T	4.08	6.93E-05	19.26%
		FIL	L	4	199,957,809	A/T	4.08	6.35E-05	18.22%
6	Chr4_203233149	TID	L	4	203,233,149	A/C	4.08	3.66E-05	18.41%
		FID	L	4	203,233,149	A/C	4.08	3.39E-05	17.16%
		TID	H	4	203,233,149	A/C	4.08	3.56E-05	16.97%
		FID	H	4	203,233,149	A/C	4.08	2.03E-05	16.31%
7	Chr4_236385528	TID	L	4	236,385,528	G/T	4.09	2.25E-05	19.25%
		FID	L	4	236,385,528	G/T	4.09	1.29E-05	18.54%
		FID	H	4	236,385,528	G/T	4.09	5.61E-05	15.46%
8	Chr5_48630086	TIL	H	5	48,630,086	C/T	5.03	4.05E-05	20.21%
		FIL	H	5	48,630,086	C/T	5.03	6.16E-05	18.07%
9	Chr5_48630116	TIL	H	5	48,630,116	A/G	5.03	4.05E-05	20.21%
		FIL	H	5	48,630,116	A/G	5.03	6.16E-05	18.07%
10	Chr5_174286151	TIL	H	5	174,286,151	C/T	5.05	1.64E-05	20.70%
		FIL	H	5	174,286,151	C/T	5.05	7.84E-05	17.30%
11	Chr8_67356036	TID	L	8	67,356,036	C/T	8.03	2.08E-05	19.89%
		FID	L	8	67,356,036	C/T	8.03	6.39E-05	17.34%
12	Chr8_130686461	TID	L	8	130,686,461	C/T	8.05	1.11E-05	20.19%
		FID	L	8	130,686,461	C/T	8.05	1.46E-05	18.59%
		TID	H	8	130,686,461	C/T	8.05	1.17E-05	18.64%
		FID	H	8	130,686,461	C/T	8.05	6.09E-05	15.52%
13	Chr9_133921410	TIL	H	9	133,921,410	C/G	9.05	4.81E-05	21.01%
		FIL	H	9	133,921,410	C/G	9.05	5.93E-05	19.14%
14	Chr10_148095509	FIL	L	10	148,095,509	A/T	10.07	2.66E-05	19.75%
		TIL	H	10	148,095,509	A/T	10.07	9.83E-05	20.05%

^a^L means low plant density, H means high plant density

^b^physical position of the SNP loci according to B73 RefGen_v2

### Candidate genes associated with significant SNPs

**Fig. 5:**
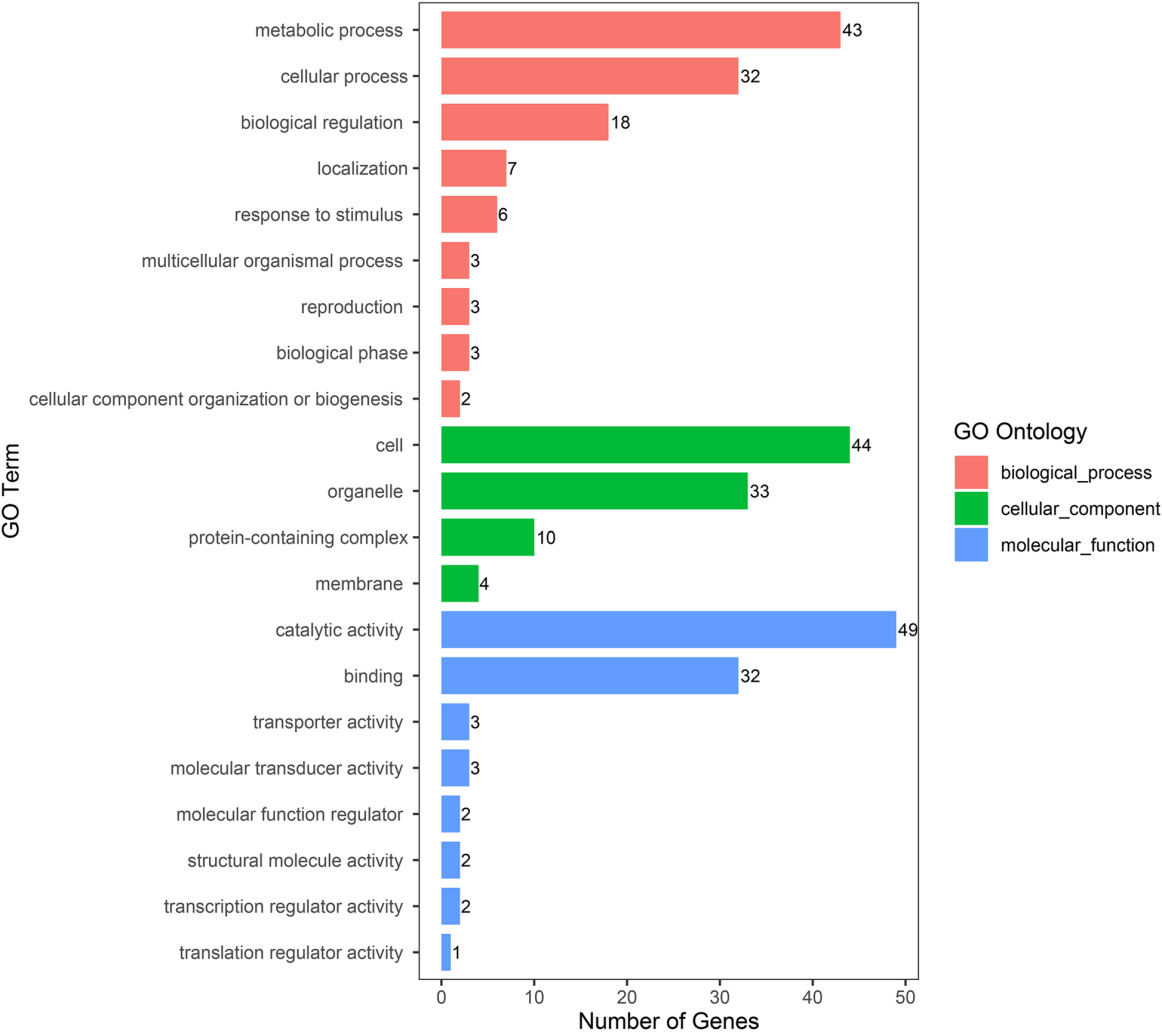
GO-second class of candidate gene

**Fig. 6:**
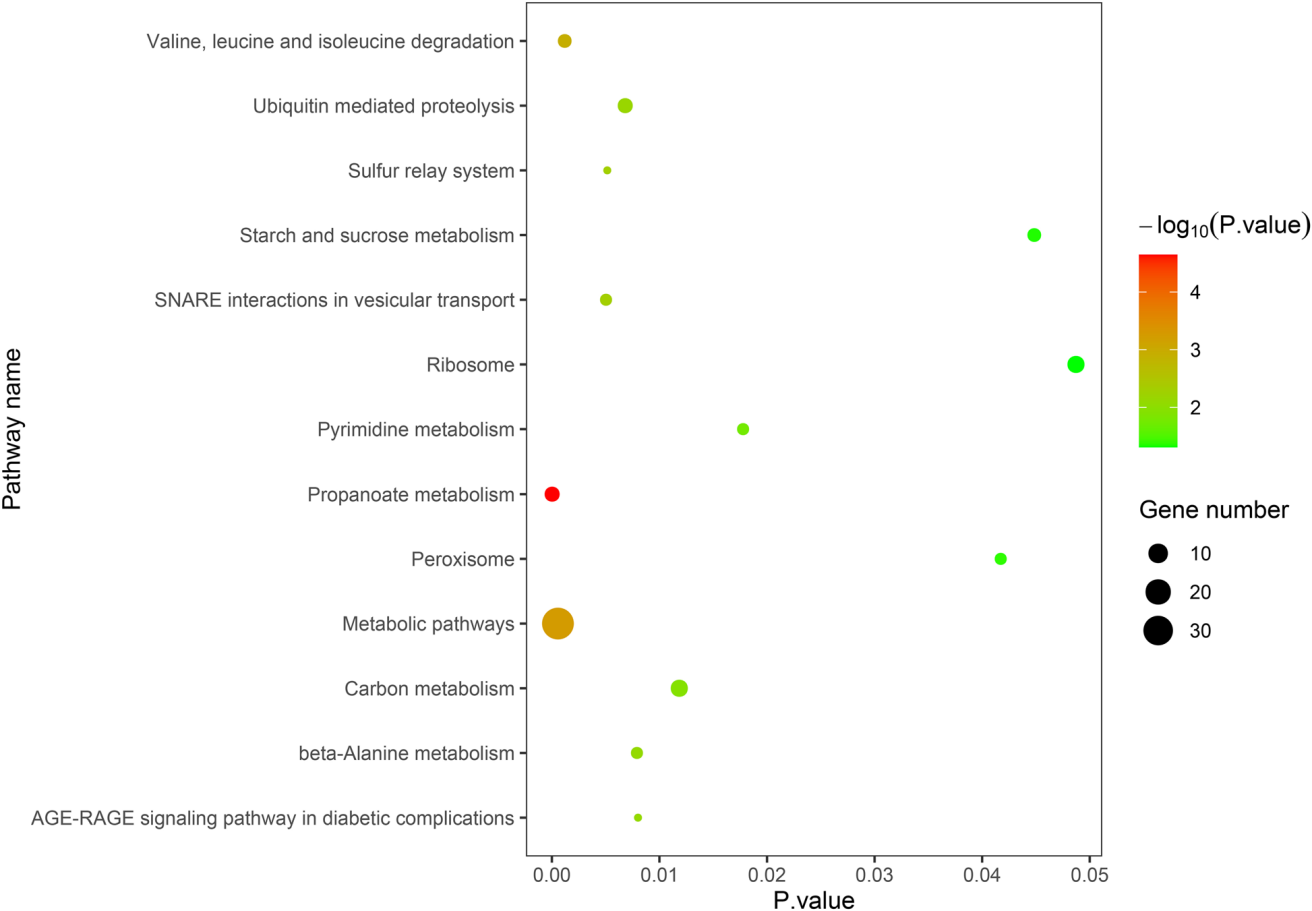
Analysis of KEGG pathway based on candidate genes (The figure was created by R version 3.6.1 based on KEGG pathway database www. kegg. jp/ kegg/ kegg1. html)

The physical locations of the SNPs were recorded using the B73 RefGen_v2 (www.maizesequence.org) based on the LD decay distance. A total of 346 candidate genes with gene descriptions were found (Table S3). The number of candidate genes involved in the six stalk lodging resistance related-traits of RPR, SBS, TIL, TID, FIL, and FID were 55, 78, 117, 37, 51, and eight, respectively. From the GO analysis results of the candidate genes in biological processes are mainly concentrated in the metabolic and cellular process, those influencing cellular component are mainly found in the intracellular and cellular anatomical entity, and those influencing molecular functions are mainly found in catalytic activity and binding (Fig. 5). As for the KEGG analysis of the candidate genes, a total of 13 pathways were identified (Fig. 6). These pathways included the carbon metabolism, ubiquitin mediated proteolysis, starch and sucrose metabolism, beta-alanine metabolism, pyrimidine metabolism, etc., which could be related to the stalk lodging. Among them, the pathway with the largest number of genes is the metabolic pathways, which have 36 candidate genes. Furthermore, we identified seven candidate genes to be associated with stalk lodging resistance (Table 5). Annotation information suggested that these candidate genes may control multiple traits during maize growth and development.

**Table 5:** Putative candidate gene of stalk lodging resistance-related traits

Trait	SNP	Bin	Candidate gene	Gene ID	RefGen_v2 Annotated Gene description
RPR	Chr6_158343036	6.06	*GRMZM2G074792*	103,630,593	probable xyloglucan glycosyltransferase
SBS	Chr1_272576164	1.1	*GRMZM2G300412*	109,942,298	UDP-glucuronic acid decarboxylase
SBS	Chr7_160255239, Chr7_160255241	7.04	*GRMZM2G072526*	100,282,931	glucan endo-1,3-beta-glucosidase
TIL	Chr5_15958677	5.03	*GRMZM2G111344*	100,381,816	UDP-glycosyltransferase
TIL	Chr10_139852648	10.06	*GRMZM2G007899*	541,747	MYB transcription factor
			*GRMZM2G311059*		
FIL	Chr2_233691559	2.09	*GRMZM2G021051*	100,217,010	gibberellin 20-oxidase
FID	Chr3_212705423	3.08	*GRMZM2G408462*	103,651,407	WRKY transcription factor

## Discussion

### Phenotypic variation, heritability, and correlations of traits

In general, obtaining an accurate measurement of phenotypic traits is essential to obtain reliable association results. The six traits investigated in this study exhibited large phenotypic variations with a normal distribution. A previous study showed that relatively high heritability will determine the power of QTL detection [33]. Our genetic analysis shows that the heritability of RPR and SBS ranged from 0.61 to 0.80. It was much higher than the range of 0.08–0.34 in a nested association population of maize [1]. The relatively high heritability in this study shows the predominant role of genetic factors for these traits.

There were significant correlations between each pair of stalk lodging resistance-related traits in this study, for instance: between RPR and SBS, which is consistent with previous results [13, 17]. Our study showed that the stalk strength traits decreased gradually with increasing density, which was consistent with previous findings [11, 34]. In the association mapping panel, a significant correlation was detected between SBS, TID, and FID. By contrast, the correlation between SBS, TIL and FIL was significantly negative, indicating that stalk strength traits are negatively associated with internode length and width at the population level. The above results suggest that some genetic factors were shared among these stalk lodging resistance-related traits.

### Mapping analysis

Compared with traditional QTL mapping, GWAS covers a wide range of genetic diversity and more allelic polymorphisms, which could exploit the short linkage disequilibrium distance and help to pinpoint the functional genes of target traits using high-density molecular markers.

Hu et al. [8] detected ten QTLs for RPR and three QTLs for Internode diameter (InD) by applying the RIL population. In this study, we used GWAS to identify some RPR-related SNPs, among which Chr7_163048364 (bin7.04) and Chr8_88680106 (bin8.03) were located in the chromosomal region with Hu et al. [8]. In addition, Chr4_203233149 (bin4.08) and Chr8_67356036 (bin8.03) for TID and FID identified by the GWAS analysis locates exactly in the interval of the InD QTLs detected by Hu et al. [8]. Liu et al. [11] identified pleiotropic QTL, *pQTL6-2*, was association with RPR, whose confidence interval encompassed 16 QTLs, its genomic region is coincided with the physical position Chr6_158343036 (158 Mb) in this study. In addition, the SNP Chr1_272576164 (272 Mb) was detected association with SBS in this study also have same physical position with Liu et al. study. The remaining SNPs in this study were first reported to be associated with lodging resistance-related traits in maize.

### Co-localization of SNPs for stalk lodging resistance traits

The SNP repeatedly detected in multiple environments is generally considered a stable SNP. Stably expressed SNPs detected in this study, five co-localized SNPs (Chr4_66017316, Chr4_16211307, Chr4_203233149, Chr4_236385528 and Chr8_130686461) were simultaneously identified under two plant densities. These stable SNPs were insensitive to the external environment and were hence considered to be important loci for the improvement of stalk lodging traits, as such, they can provide references for further gene cloning. Meanwhile, some specific SNPs were detected at high or low plant densities, respectively, which may be environmentally-specific loci requiring further genetic mapping.

From the comparison, we found some co-located locus in different densities in the same environment, but extremely few stable sites in different environments. The reason we detected less consistent loci in different environments may be because stalk strength trait itself is a relatively complex quantitative trait and is greatly affected by the environment. In addition, we found that the heritability of these traits is relatively low. This reason was further confirmed. From the results of the phenotypic correlation analysis, the correlation coefficient of both TID and FID was as high as 0.97 at both densities. Similarly, we located three SNPs (Chr4_16211307, Chr4_203233149, Chr8_130686461) associated with both TID and FID at both densities, this confirms the views of previous, phenotypic correlations between quantitative traits may derive from the correlation between QTL controlling them [35]. However, there were a large number of SNPs that did not co-located, indicating that lodging-related traits in maize seem to be controlled not only by several major QTLs but also by multiple micro-effect QTLs in specific locations or environments [36].

### Candidate genes analysis

We identified 346 candidate genes in total located around common loci for stalk lodging resistance-related traits, which are involved in a variety of biochemical metabolic pathways. Based on the information of the gene model on MaizeGDB (Table S3), seven potential candidate genes related to RPR, SBS, TIL, FIL and FID were obtained (Table 5). Notably, some candidate genes correlated to stalk lodging-related traits were related to cellulose and lignin biosynthesis, essential for the cell wall development in the plant stem. For instance, beta-amylase (AMY), beta-glucosidase (GLU), UDP-glycosyltransferase (UGT), and protein kinase played an essential role in the synthesis of cell wall components [37]. Indeed, modify the expression of a transcription factors by changing the mRNA abundance of downstream target genes to change the biosynthesis of lignin and he lodging resistance of stalk can be increased [38]. Interestingly, seven candidate genes were found to be related to cell wall components in this study (Table 5). *GRMZM2G074792*, which is located in Chr6_158343036 of RPR, encodes xyloglucan glycosyltransferase and related to plant cell wall cellulose synthesis, which is the major source of cellulose-harbours enzyme [39]. *GRMZM2G300412*, encoded for UDP-glucuronic acid decarboxylase, was located in Chr1_272576164 of SBS, involving in metabolic pathways and amino sugar and nucleotide sugar metabolism. *GRMZM2G072526* was located in Chr7_160255239 and Chr7_160255241, controlling SBS, whose encoded glucan endo-1,3-beta-glucosidase is mainly involved in carbohydrate metabolism, it is associated with cell wall synthesis, which may be related to maize lodging. Previous studies demonstrated that UDP-glucuronic acid decarboxylase was a key enzyme in the synthesis of UDP-xylose for the formation of xylans during cell wall biosynthesis [40]. *GRMZM2G111344*, was located in Chr5_15958677 of TIL, encoding for UDP-glycosyltransferase (UGT), involved in flavonoid biosynthesis and biosynthesis of secondary metabolites. According to previous studies, UGT was the key precursors of cell wall carbohydrates [37]. These descriptions indicate that regulation of the expression of these genes may affect cell wall formation. The candidate genes *GRMZM2G007899* and *GRMZM2G311059*, were located in Chr10_139852648 of TIL, showed high expression of MYB transcription factor had increased ectopic lignin and the xylem vessels were regular and open, are related transcriptional activators of the lignin biosynthetic pathway during secondary cell wall formation in *Arabidopsis* [41, 42]. In rice, *GRMZM2G021051* was located in Chr2_233691559 of FIL, whose the homologous with shortened basal internodes, is a new rice lodging-resistance gene and encodes a gibberellin (GA) 2-oxidase and can control the elongation of internodes at the base of the stem by regulating the activity of the GA [43]. *GRMZM2G408462*, which is located in Chr3_212705423 of FID, encoded for WRKY transcription factor, whose directly regulate expression of the major monolignol biosynthetic genes and genetic modification of genes involved in lignin biosynthesis [44, 45]. Although the role of these genes in maize requires further investigation, they should be used as target sites for the development of maize lines resistant to lodging.

## Conclusion

In this study, we identified 6, 3, 10, 8, 8, 7 SNPs associated with RPR, SBS, TIL, TID, FIL, FID at low plant density and 5, 9, 7, 5, 7, 6 SNPs associated with RPR, SBS, TIL, TID, FIL, FID at high plant density, respectively, via GWAS. Most markers were located within or close to QTLs identified in previous studies. We were particularly interested in the seven potential candidate genes that were predicted based on functional annotations, but further investigation is needed for verification of this hypothesis. These findings shed light on the genetic basis of six stalk lodging resistance related-traits, and candidate genes could be used for further positional cloning.

## Materials and methods

### Plants materials and field experiments

A total of 248 diverse maize inbred lines were used to form an association mapping panel. All lines were grown according to the split-plot set two densities, two replicates for each density, and a low density of 75,000 plants/ha and a high density of 105,000 plants/ha. The work was performed at the Experimental Station of Hebei Agricultural University in Baoding and Shijiazhuang in 2015 and 2016. For each replicate, each line was grown in a 3-m long single-row plot, with a 0.6-m inter-row spacing. All of the plant materials used in our study were derived from the China Agricultural University and National Maize Improvement Center of China.

### Phenotype evaluation

Based on previous studies on stalk lodging resistance in maize, we decided to measure morphological traits and stalk strength during one week after grain filling [11]. Five representative plants of each line from each replicate were selected for evaluation and the mean values for each line were computed for each trait. The TIL, FIL, TID, and FID were measured using electronic micrometers. At the same time, morphological characters were measured with the same material, RPR and SBS were measured in the middle of the flat side of the third and fourth internode of the stalk using a stalk strength appliance YYD-1 (Zhejiang TopuYunnong Science and Technology Co., Ltd, Zhejiang, China). At the base of the stem, the middle part of the third and fourth internodes is inserted at a constant speed and perpendicular to the direction of the stem, and the maximum penetration of the stem epidermis is read. Similarly, the bending strength of the stalk is also pressed at the center of the stalk at a uniform speed, and the force should not be too strong and record the value. The range of measurement was between 5 and 500 N, with a resolution of 0.1 N; reported units of RPR and SBS are in N/mm^2^ and N, respectively.

### Statistical analysis of phenotypic data

The mean value of each inbred line for each trait was used for descriptive statistical analysis. Analysis of variance (ANOVA) was carried out with SPSS19.0 for related traits of stalk lodging resistance under two plant densities in the association mapping panel. Broad-sense heritability (*h*^*2*^_*B*_) was calculated according to Knapp et al. [46].

$${h}_{B}^{2}={\sigma }_{g}^{2}/({\sigma }_{g}^{2}+{\sigma }_{ge}^{2}/e+{\sigma }_{\varepsilon }^{2}/re)$$

where ${\sigma }_{g}^{2}$ is the genetic variance, ${\sigma }_{ge}^{2}$ is the interactive effect of genotype × environment, ${\sigma }_{\varepsilon }^{2}$ is the error variance, *e* is the number of environments, and *r* is the number of replications in a given environment.

The best linear unbiased prediction (BLUP) of the phenotypic values of each line was calculated across all environments using the R package “lme4” [47]. The BLUP value of each line was used for the GWAS analysis. The correlation analysis was performed using the “Performance Analytics” package in R.

### Genotyping

The GBS method was used to genotype the 248 inbred lines of the association panel [29]. First, the genomic DNA was extracted from leaves of maize under normal growth conditions using the cetyltrimethylammonium bromide (CTAB) method [48]. The DNA concentration and integrity were measured with NanoDrop 2000 instrument (Thermo Fisher Scientific, Waltham, MA, USA) and agarose gel electrophoresis, respectively. The extracted DNA of each line was digested using the restriction enzyme ApeKI and ligated with bar code. The DNA samples of certain numbers were mixed, purified, amplified, purified again, and chosen according to fragment length. Those fragments were evaluated using the length test, Paired-End-Tag by Illumina Hiseq2000. Then selected sequences were aligned to the B73 reference genome (the second version) using the BWA software, followed by SNP calling using Samtools [49]. SNPs with a missing rate < 0.2 and minor allele frequency (MAF) > 0.05 were selected. Finally, a total of 83,057 SNPs were used for the GWAS analysis. The PLINK 1.90 beta software was used to estimate LD between pairs of SNPs within 200 kb in the genomic region based on the Hill and Weir method [50, 51]. The LD decay distance for this association mapping panel was 120 kb (r^2^ = 0.1) based on previous study [52, 53]. The population structure (Q) was estimated using the software Admixture 1.3, while kinship (K) was estimated using Analysis-Kinship in Tassel 5.0.

### Genome-wide association studies

GWAS data was analyzed with the mixed linear model (MLM) using the “GAPIT” package in R. The SNP markers of six stalk lodging resistance related-traits in the association mapping panel together with the Q and K matrix were used as covariates to decrease spurious association and detect marker loci combining with target traits. The GWAS analysis is performed with a Bonferroni correction, however this was found to be too strict for less significant trait associations. Therefore, we reduced the significance threshold to–log10 (P) ≥ 4 for all traits [28].

### Prediction of candidate genes

The candidate gene analysis was based on the maize inbred line B73 reference genome version v2 (centering on the marker site and extending 120 kb upstream and downstream) and searching for the information and functions of the candidate genes on the MaizeGDB genome browser (http://www.maizegdb.org/) and NCBI website (https://www.ncbi.nlm.nih.gov/). Gene ontology (GO) enrichment analysis was performed using the Gene ontology website (http://www.geneontology.org/). Kyoto Encyclopedia of Genes and Genomes (KEGG) pathway enrichment analysis was performed using the KOBAS version 3.0 (http://kobas.cbi.pku.edu.cn/kobas3/?t=1) [54].

## Supplementary Information

**Additional file 1: Supplementary Table S1.** List of genes within the 120 kb upstream and downstream extension of significant SNPs identified via GWAS. **Supplementary Table S2.** SNPs detected for lodging resistance-related traits using BLUP value in the association mapping panel. **Supplementary Table S3.** List of genes within the 120 kb upstream and downstream extension of significant SNPs identified via GWAS.

## Data Availability

All data generated or analyzed during this study are included in this published article and its supplementary information files.

## Abbreviations

RPR: Rind penetrometer resistance;
SBS: Stalk buckling strength
FIAG: Fourth internodes above ground
QTL: Quantitative trait loci
RIL: Recombinant inbred line
SNP: Single nucleotide polymorphisms
GWAS: Genome-wide association study
GBS: Genotyping by sequencing
TIL: Third internodes length
FIL: Fourth internode length
TID: Third internode diameter
FID: Fourth internode diameter
ANOVA: Analysis of variance
BLUP: Best linear unbiased prediction
MAF: Minor allele frequency
GO: Gene ontology
KEGG: Kyoto encyclopedia of genes and genomes
CV: Coefficients of variation
InD: Internode diameter
AMY: Beta-amylase
GLU: Beta-glucosidase
UGT: UDP-glycosyltransferase
MLM: Mixed linear model
CTAB: Cetyltrimethylammonium bromide
